# Conductive and transparent multilayer films for low-temperature TiO_2_/Ag/SiO_2_ electrodes by E-beam evaporation with IAD

**DOI:** 10.1186/1556-276X-9-35

**Published:** 2014-01-16

**Authors:** Po-Kai Chiu, Chao-Te Lee, Donyau Chiang, Wen-Hao Cho, Chien-Nan Hsiao, Yi-Yan Chen, Bo-Ming Huang, Jer-Ren Yang

**Affiliations:** 1 Instrument Technology Research Center, National Applied Research Laboratories, Hsinchu Science Park, Hsinchu 300, Taiwan; 2 Institute of Materials Science and Engineering, National Taiwan University, Taipei 10617, Taiwan

**Keywords:** TiO_2_/Ag/SiO_2_, E-beam, Ion-beam-assisted deposition, Room temperature, Transparent conducting oxides

## Abstract

Conductive and transparent multilayer thin films consisting of three alternating layers (TiO_2_/Ag/SiO_2_, TAS) have been fabricated for applications as transparent conducting oxides. Metal oxide and metal layers were prepared by electron-beam evaporation with ion-assisted deposition, and the optical and electrical properties of the resulting films as well as their energy bounding characteristics and microstructures were carefully investigated. The optical properties of the obtained TAS material were compared with those of well-known transparent metal oxide glasses such as ZnO/Ag/ZnO, TiO_2_/Ag/TiO_2_, ZnO/Cu/ZnO, and ZnO/Al/ZnO. The weathering resistance of the TAS film was improved by using a protective SiO_2_ film as the uppermost layer. The transmittance spectra and sheet resistance of the material were carefully measured and analyzed as a function of the layer thickness. By properly adjusting the thickness of the metal and dielectric films, a low sheet resistance of 6.5 ohm/sq and a high average transmittance of over 89% in the 400 to 700 nm wavelength regions were achieved. We found that the Ag layer played a significant role in determining the optical and electrical properties of this film.

## Background

The conduction electrons in a metal behave like a gas of nearly free electrons. Radiative surface modes can be excited at the boundary of the metal by using non-normal incident p-polarized light. In an effort to produce conductive and transparent substrates, multilayer coatings of the type dielectric material/metal/dielectric material (DMD) have been developed, as exemplified by ZnS/Ag/ZnS, ZnO/Ag/ZnO, ITO/Ag/ITO, and ITO/CuAg/ITO (ITO, indium-tin oxide) [1-4]. These transparent multilayer films, which have low reflectance, good transparency, and high conductivity when prepared by low-temperature sputtering deposition, have been studied for developing functional materials such as heat mirrors [5,6]. The transmittances at 550 nm and the sheet resistances of various multilayer cathodes are shown in Table 1. The material composed of TiO_2_/Ag/TiO_2_ (TAT) exhibited a transmittance of 68%, whereas that composed of SiO_2_/Ag/SiO_2_ (SAS) exhibited a transmittance of 67%. The light pathway due to multiple reflections leads to a slight decrease in the transmittance of the multilayer [7-9]. The specific resistivity of the metal layer can be calculated by assuming that the total resistance of the material results from the individual resistance of the three single layers coupled in parallel. This is shown in the equation below.

$$\frac{1}{R_{\mathrm{total}}}=\frac{1}{R_{{\mathrm{SiO}}_{2}}}+\frac{1}{R_{\mathrm{Ag}}}+\frac{1}{R_{{\mathrm{SiO}}_{2}}}$$

**Table 1:** Transmittances and sheet resistances of various cathodes

**Conditions**	**Percentage of**	**Sheet**
	**transmittance 550 nm**	**resistance (Ω cm)**
A1 (20 nm)	~45	13
SiO_2_/Ag/SiO_2_ (40:10:40 nm)	~67	2.93
ZnO/Cu/ZnO (58:10:63 nm)	~74	17
ZnO/Cu/ZnO (40:10:40 nm)	~70	17
ZnO/A1/ZnO (40:10:40 nm)	~62	40
TiO_2_/Ag/TiO_2 _(40:10:40 nm)	~68	0.7
ZnO/Ag/ZnO (40:10:40 nm)	~90	5

This assumption is justified if the film boundary effects are negligible [7-9]. Silver was found to perform the best as the middle metal layer in sandwiched DMD structures. A pure Ag metal film has the lowest resistivity of all metals and exhibits relatively low absorption in the visible region. The optical and electrical properties of DMD films can be adjusted to achieve various transmittances with a peak in the spectra by suitably varying the thickness of the Ag layer. TiO_2_, a dielectric material, is used in the DMD structure because of its high refractive index, good transparency in the visible region, and easy evaporation. SiO_2_ is very stable and can be used as a protective layer on top of the Ag surface to avoid the deterioration of the properties of the metal during exposure to certain environmental conditions. Ag, SiO_2_, and TiO_2_ are also materials that are most frequently used in the fabrication of optical and electrical devices at a relatively low cost. This can be achieved by thin film deposition, applying either evaporation or sputtering methods under normal vacuum conditions.

In the case of SAS material, a minimal current seems to flow into the device because of the low conductivity and charge densities for current flow observed within it. However, Kim and Shin [10] reported conductivity enhancement achieved by introducing zinc cations into the amorphous silica layer. This means that we can obtain better current injection into the transparent organic light-emitting diodes by properly treating SAS cathodes. Such cathodes exhibit two separate mechanisms for resonant tunneling current injection: one for the low-voltage region and one for transparent conducting oxides (TCOs) currents for the high-voltage region.

In this study, multilayer transparent conductive coatings (DMD) were fabricated for low-temperature-sintered electrodes containing mesoporous TiO_2_. This compound was chosen as one of the dielectric materials because of its suitable properties as described above. SiO_2_ was chosen as the other dielectric material, since it also has a low refractive index and exhibits good transparency in the visible region. In addition, this semiconductor is very stable, as mentioned before, and can be easily evaporated. Finally, Ag was chosen as the conductive layer because of its suitable optical properties in the visible region. Hence, TiO_2_/Ag/SiO_2_ (TAS) transparent films were fabricated, and their possible application in TCOs was examined.

## Methods

### Fabrication of TiO_2_/Ag/SiO_2_ transparent films

#### Deposition techniques

TAS multilayers were fabricated by electron-beam (E-beam) evaporation with ion-assisted deposition ion-beam-assisted deposition (IAD) under a base pressure of 5 × 10^−7^ Torr. The substrates were kept at room temperature before starting deposition. The working pressure for the deposition of the first layer (TiO_2_) was maintained at 4 × 10^−4^ Torr with O_2_, whereas the deposition of the third layer (TiO_2_) was maintained at 6 × 10^−6^ Torr (without O_2_) in the 0- to 10-nm thickness range and at 4 × 10^−4^ Torr (O_2_) in the 10- to 70-nm thickness range. The working pressure for the deposition of the second layer (Ag) was maintained at 6 × 10^−6^ Torr (without O_2_). The deposition rate of TiO_2_ was 0.3 nm/s and that of Ag was 0.5 nm/s. The ZnO film was bombarded by oxygen ions with ion beam energies of 400 to 500 W, whereas the Ag film was bombarded by argon ions with ion beam energies of 400 to 500 W. The film thickness was determined using an optical thickness monitoring system, and the evaporation rate was deduced from the measurements of a quartz oscillator placed in the deposition chamber. The thicknesses of the glass-attached TiO_2_ layer, Ag layer, and protective layer SiO_2_ were determined using the Macleod simulation software.

### Optical properties, electrical properties, and microstructure analysis

Optical transmittance measurements were performed on the TAS multilayers using an ultraviolet–visible-near-infrared (UV–vis-NIR) dual-beam spectrometer in 400 to 700 nm wavelength range. Optical polarization was applied to the single films by ellipsometric measurements to increase the refraction index. The crystal orientation of the deposited films was examined by x-ray diffraction (XRD) with Cu Kα radiation. A transmission electron microscope (JEOL 2000 EX H; JEOL Ltd., Akishima, Tokyo, Japan), operated at 200 kV, and a field-emission gun transmission electron microscope, operated at 300 kV, were used for cross-sectional microstructure examination. Energy-dispersive spectra (EDS) and electron diffraction patterns obtained using this equipment enabled detailed sample characterization. The sheet resistance of the samples was measured by a Hall system. X-ray photoelectron spectroscopy (XPS) measurements were carried out using a Thermo Scientific K-Alpha spectrometer (Thermo Fisher Scientific, Hudson, NH, USA). An Al x-ray at 1,487 eV was used as the light source, and the peak positions were internally referenced to the C 1*s* peak (arising from the methylene groups of dodecanethiolate) at 284.9 eV [11]. All the binding energies are referenced to the clean Ag 3*ds*/2 peak at 368.22 eV.

## Results and discussion

### Film structure

**Figure 1:**
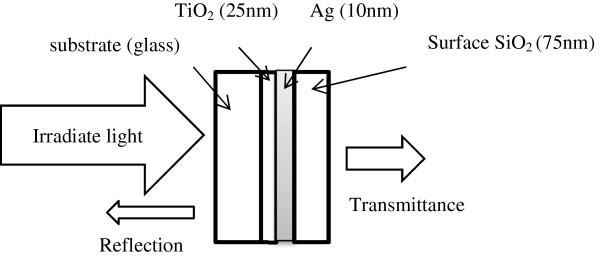
**Structure of the transparent film (TiO**_**2**_**/Ag/SiO**_**2**_**, TAS).** Each layer was fabricated by E-beam evaporation with IAD.

A multilayer thin-film structure with maximum transmittance can be designed using the Macleod simulation software. The admittance diagram of a three-layer TAS film structure allows us to determine the optimal thickness of each layer. The function of the Ag layer, which should be thick to achieve good conductivity, is mainly to filter UV and IR light; on the other hand, the TiO_2_ and SiO_2_ films are expected to increase the transmittance of visible light. Sawada et al. [12] highlighted that a 10-mm-thick Ag layer led to fewer variations in the sheet resistance, and the transmittance was inversely proportional to the thickness of the metal layer. The optimal thickness of the Ag layer was found to be 10 mm. The thickness of the bottom TiO_2_ layer should be in the range of 20 to 25 nm and that of the top protective layer in the range of 65 to 75 nm (these are the best values to reduce the distance of equivalent admittance and air admittance). Minimal reflection conditions can be achieved by considering these restrictions. In this way, we calculated the value of yE for different thicknesses of the TiO_2_ and SiO_2_ films (Table 2). Figure 1 shows the structure of the studied multilayer film: substrate/TiO_2_/Ag/SiO_2_/air.

**Table 2:** **Optical spectra of a substrate TiO**_**2**_**/Ag/SiO**_**2**_**/air structure simulated using the Macleod software**

**Value of yE (Tio**_**2**_**/Ag/SiO**_**2**_**)**	**Re (admittance)**	**Im (admittance)**
20:10:20 nm	0.87	−1.42
40:10:40 nm	0.78	−0.98
60:10:60 nm	0.66	−0.78
20:10:40 nm	0.6	−0.95
25:10:70 nm	0.7	−0.40

### Crystallinity

**Figure 2:**
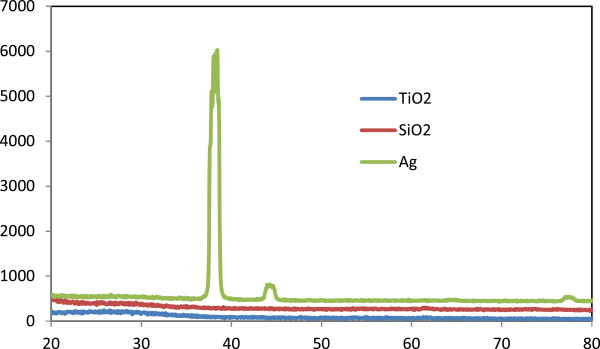
**XRD patterns of TiO**_**2 **_**and SiO**_**2 **_**thin films fabricated on glass.** XRD patterns showing that the TiO_2_ and SiO_2_ thin films fabricated on glass by E-beam evaporation with IAD exhibit a preferential amorphous growth.

Figure 2 shows the XRD patterns obtained for the multilayer structure deposited by E-beam evaporation with IAD at room temperature. As seen in the XRD patterns, the TiO_2_ and SiO_2_ thin films evaporated on glass (an amorphous substrate) preferred to grow amorphously. A peak corresponding to crystalline Ag was also clearly visible, showing preferred growth of the metal in the (111) direction. This might be the result of using a high-momentum ion beam, since such beams can increase the evaporation rate and decrease the amount of Ag that is oxidized.

### Optical spectroscopy of the conductive and transparent films

**Figure 3:**
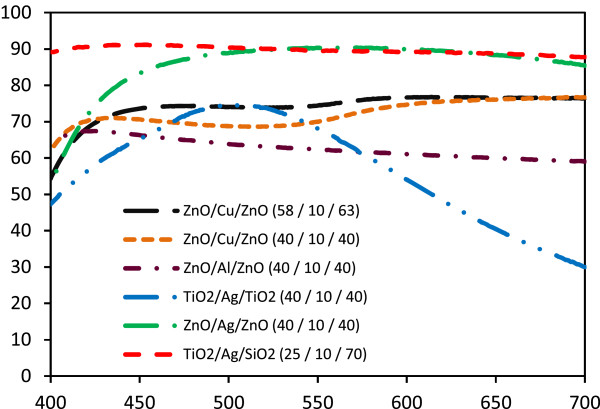
Transmittance spectra of DMD structures with different metal and dielectric layers.

**Figure 4:**
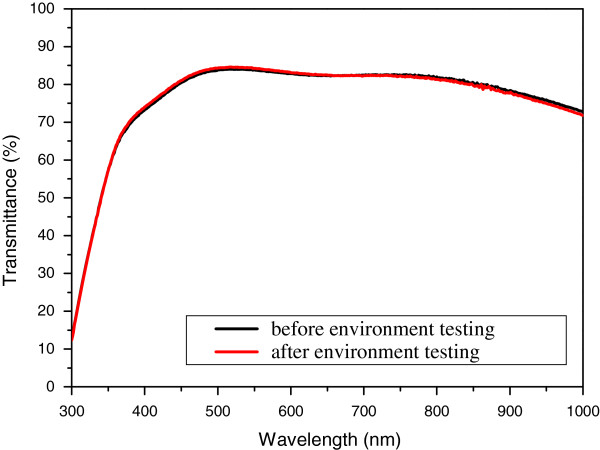
Transmittance values before and after environmental testing.

Figure 3 shows the transmittance spectra of several coatings. The TAS film has a layer-wise thickness of 25:10:70 nm. The thickness of the Ag layer was found to affect the transmittance of the incident light from the glass substrate, which decreased gradually with increasing thickness. If the materials of the first and last layers have respective higher and lower refractive indices than that of the substrate, the transmittance of short-wavelength radiation increases, and the other wavelengths retain the same transmittance in the visible region. Figure 4 shows the transmission spectra of the transparent film measured before and after environmental testing. After the tests were carried out at 55°C and 95% moisture for 6 h (ISO 9211), the transmittance of the TAT multilayers decreased, whereas no attenuation of visible light was observed for the TAS multilayers. This shows that the SiO_2_ film acted as a very good moisture barrier material, thereby preventing transmittance losses in the system. The transmittance of the TAS film improved with decreasing reflectance, which is related to the high-reflection index of the TiO_2_ layer. The weathering resistance of the TAS film could be improved by using a protective SiO_2_ film as the uppermost layer.

### Microstructure of the TAS multilayers

**Figure 5:**
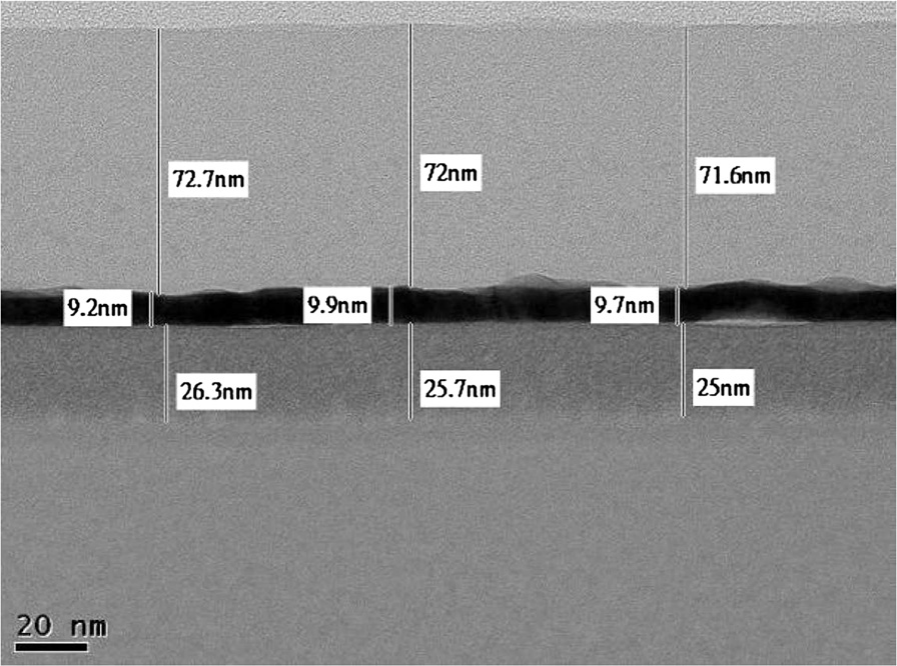
TEM image of the cross section of a TAS film.

**Figure 6:**
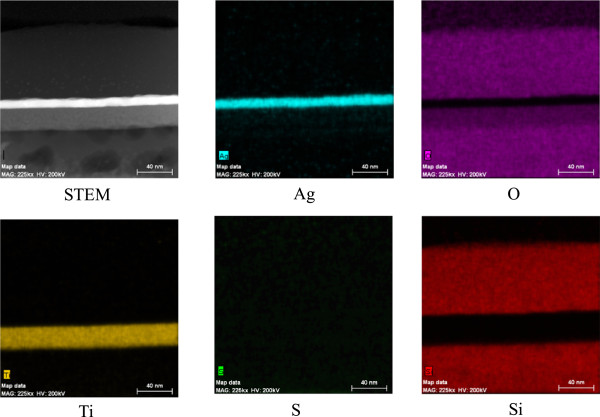
Cross-sectional STEM mapping of TAS multilayer structures deposited by E-beam evaporation with IAD.

**Figure 7:**
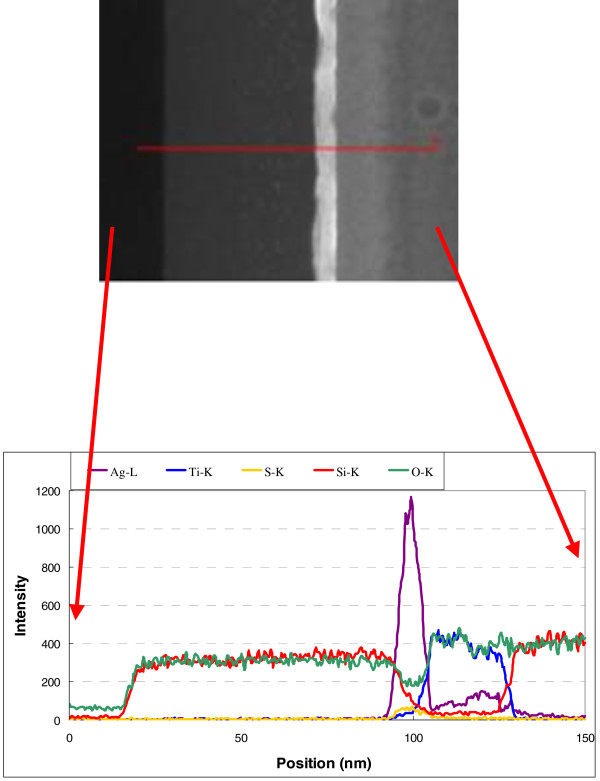
**EDS line scans of TiO**_**2**_**/Ag/SiO**_**2 **_**multilayer structures deposited by E-beam evaporation with IAD.**

The transmission electron microscopy (TEM) image of the cross section of a TAS film on a glass substrate presented in Figure 5 confirms that each layer (TiO_2_, SiO_2_, and Ag) had a flat and smooth structure, which suggests high conductivity at the Ag layer of the TAS film. The transparent conductive multilayers (TAS) fabricated by E-beam coating with IAD have lower resistance than those prepared without IAD [2]. This is due to the different morphologies of the Ag layers. The film prepared without IAD exhibits an island structure, and the low contact between the Ag islands results in a higher resistivity. On the other hand, the Ag layer prepared by with IAD is smooth and has a low resistivity. The TAS film reported herein was prepared by E-beam coating with IAD and has a low resistivity (sheet resistivity of 6.5 Ω/sq for a 9.5-nm-thick Ag layer). The Ag layer in this material is flat and sufficiently smooth to make it attractive for use as a transparent film. The film thicknesses determined from the TEM images are consistent with those predicted by simulations carried out using the Macleod software. The 10-nm-thick Ag layer was a continuous strip exhibiting a nanoscale crystalline structure. While the TiO_2_ films were also polycrystalline, the SiO_2_ films exhibited an amorphous structure. The EDS mapping images shown in Figure 6 suggest that no oxides are present in the Ag layer, although diffusion is possible. Figure 7 shows EDS line scans that confirm the results of EDS mapping. The formation of partial nanocrystals is also clearly visible.

### AFM topographic analysis

**Figure 8:**
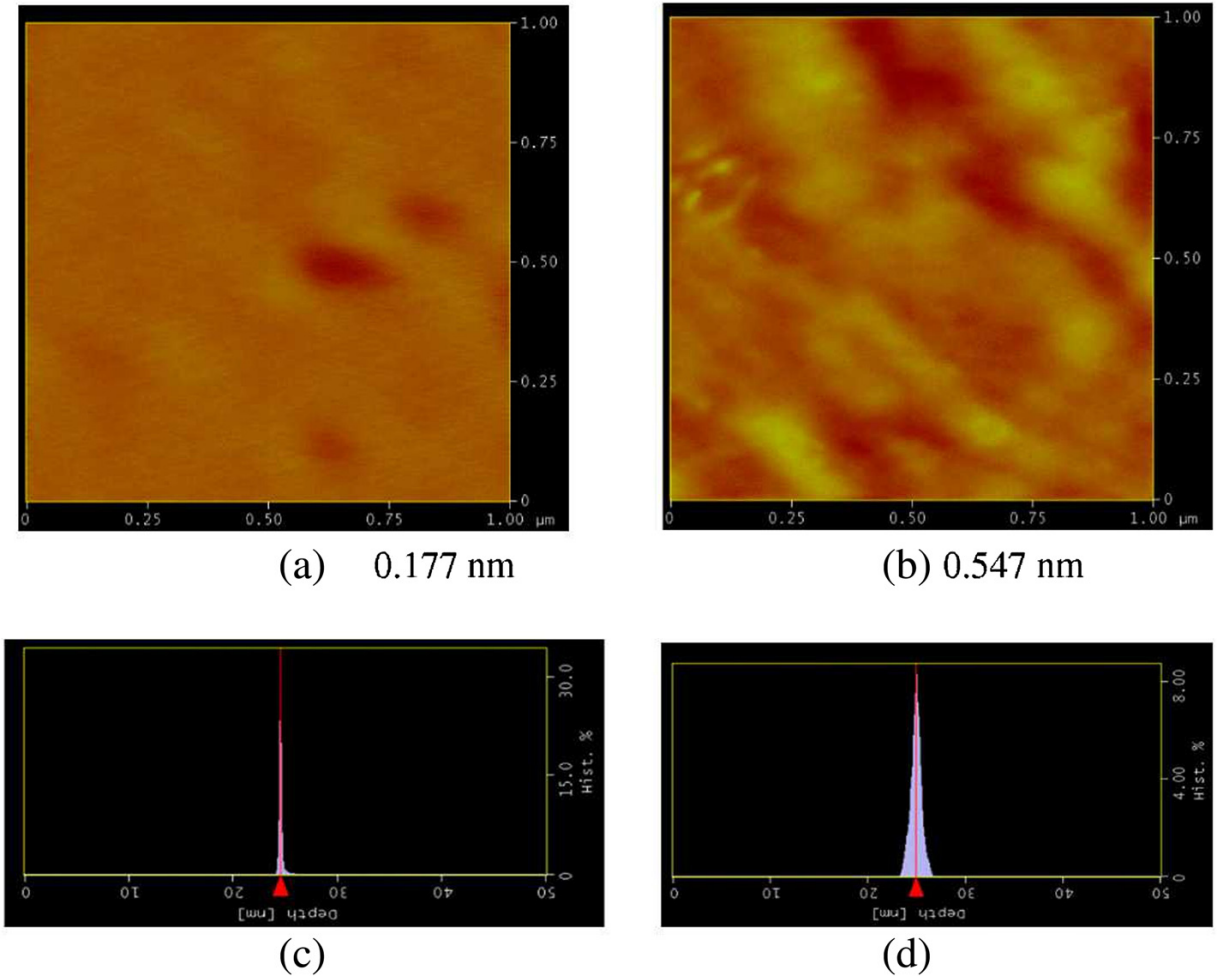
**AFM topography images of (a) an ultra-smooth, thin Ag film on glass (B270) and (b) an uncoated glass substrate (B270). (c,d)** Histograms (2D surface height values) obtained from the respective topography images.

Figure 8 shows a representative atomic force microscopy (AFM) topographic image of a silver film fabricated by E-beam coating with IAD after optical monitored ion etching treatment. Figure 8a presents the 10-nm-thick Ag film deposited on glass, whereas Figure 8b shows an image of the uncoated substrate. Two-dimensional histograms containing surface height values determined from the respective topographies are also shown. The obtained Ag film exhibited a root-mean-square (RMS) roughness of 0.177 nm. The images (1 μm × 1 μm or 512 × 512 pixels) were automatically plane-fitted (to compensate for any sample tilts), and a color scale was used to represent the height distribution. The *Z* axes of the height histograms were scaled relative to the peak height. In addition, the surface of the evaporated Ag/glass film usually had an RMS roughness above 5 nm [13], which is an order of magnitude greater than that for the optical monitored ion etching treated E-beam coating with IAD films.

### Electrical properties

The ideal work function of Ag is 4.4 eV, which is smaller than that of TiO_2_ (4 to 6 eV) [14] and higher than that of SKh (3.03 to 3.41 eV) [15]. When two layers are in contact with each other, the Fermi levels align in equilibrium by the transfer of electrons from Ag to SiO_2_ and TiO_2_. The electrical properties of the system improve under these conditions. In this case, there is no barrier for the electron flow between Ag and SiO_2_, which means that the electrons can easily move from the Ag layer to the SiO_2_ layer. According to Schottky’s theory, we expect high carrier concentrations in multilayer TAS films.

### X-ray photoelectron spectroscopy

**Figure 9:**
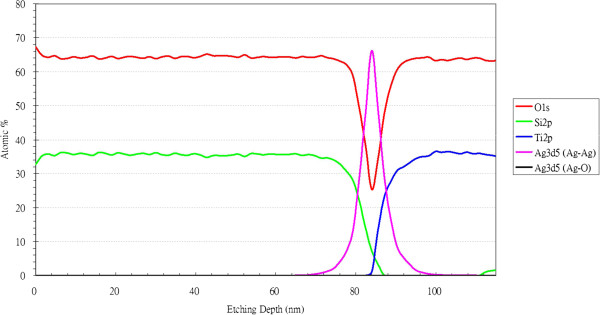
Relationship between atomic percentage and etching depth, determined by XPS analysis.

**Figure 10:**
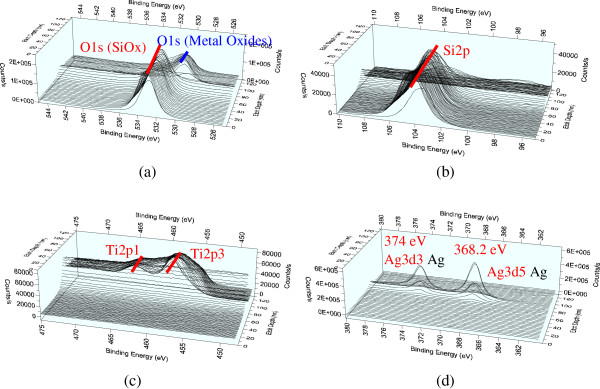
**XPS analysis of the bonds. (a)** The oxide bond. **(b)** The Si-O bond of SiO_2_. **(c)** The Ti-O bond of TiO_2_. **(d)** The Ag-Ag bond.

Figures 9 and 10 show the XPS spectra of a TAS sample in the Si 2*p*, Ti 2*p*, O 1*s*, and Ag 3*d* regions. The same TiO_2_, SiO_2_, and silver peaks have also been clearly identified for other bimetallic clusters, revealing that our multilayer samples are composed of stable titanium oxide and silicon oxide films and contain pure Ag atoms. The observed peak positions are very close to those reported for ideal vacuum-evaporated TiO_2_, SiO_2_, and silver films, with the differences (including those between the 3*d*5/2 and 3*d*3/2 peaks for silver, 6.0 eV) also being exactly the same as the handbook values reported for zero-valent silver [16]. This observation suggests that most of the silver atoms in the TAS multilayers are in the zero-valent state. One would expect that a significant amount of the outer metal atoms is oxidized from Ag^0^ to Ag^+1^ upon thiolate formation, with a shift of the Ag 3*d*5/2 peak to higher binding energies (by 0.7 to 0.9 eV).

## Conclusions

E-beam evaporation with IAD has been applied to produce TAS layers with favorable properties: the sheet resistivity of the obtained material was 6.5 Ω/sq and its average transmittance (400 to 700 nm) was 89%. Environmental testing under high temperature and humidity conditions demonstrated that the amorphous SiO_2_ layer was stable and could avoid silver oxidation and vulcanization. The resulting thickness and structure of the Ag layer were the main factors determining the electrical and optical properties of the multilayer structures. According to the results of both optical design and simulations, the first layer was fabricated using a high-reflection-index material, whereas the last layer was fabricated using a low-reflection-index material. This structure was introduced to maximize the average transmittance of visible light.

## Competing interests

The authors declare that they have no competing interests.

## Authors' contributions

PKC, DC, CNH, and JRY designed the experiment and measurements. CTL, WHC, YYC and BMH executed the experiments. CNH and JRY examined the written report. All authors read and approved the final manuscript.
